# Mixed methods study on the feasibility of implementing periodic continuous glucose monitoring among individuals with type 2 diabetes mellitus in a primary care setting

**DOI:** 10.1016/j.heliyon.2024.e29498

**Published:** 2024-04-16

**Authors:** Unn-Britt Johansson, Sissel Andreassen Gleissman, Maarit Korkeila Liden, Marie Wickman, Berit Gustafsson, Stefan Sjöberg

**Affiliations:** a Department of Health Promoting Science, Sophiahemmet University, P.O. Box 5605, SE-114, 86, Stockholm, Sweden; b Department of Clinical Science and Education, Södersjukhuset, Karolinska Institutet, P.O. Box, 5605, SE-114 86, Stockholm, Sweden; c Department of Nursing Science, Sophiahemmet University, P.O. Box 5605, SE-114 86, Stockholm, Sweden; d Insurance Clinic, Sophiahemmet, P.O. Box 5605, SE-114 86, Stockholm, Sweden

**Keywords:** Type 2 diabetes, Continuous glucose monitoring, Feasibility: focus group interview: person-centered care

## Abstract

**Background:**

Health care professionals (HCPs) play a central role in leveraging technologies to support individuals with diabetes. This mixed-method study was completed to determine the feasibility of implementing periodic continuous glucose monitoring (CGM) in a primary care setting.

**Aim:**

This study aimed to evaluate and describe the experiences of using periodic CGM with data visualization tools in patients with type 2 diabetes to foster a person-centered approach in a primary care setting.

**Methods:**

Fifty outpatients aged ≥18 years, diagnosed with type 2 diabetes, and with a disease duration of at least 2 years were included in this study. Data were collected from April 2021 to January 2022. Patients completed a single period of sensor measurements for 28 days and a diabetes questionnaire about feelings and experiences of health care. HbA1c was also measured. A focus group interview was conducted to evaluate and describe the HCPs experiences of using periodic CGM.

**Results:**

Patients reported to HCPs that the CGM device was comfortable to wear and noted that LibreView was easy to use when scanning the sensor to obtain and visualize the glucose levels and trends. Data availability of CGM data was >70 %.

Clinical observations revealed a mean reduction in HbA_1c_, mmol/mol from 60.06 [7.65 %] at baseline to 55.42 [7.20 %] after 4 weeks (p < 0.001). Two categories were identified: 1) Fostering dialogue on self-care and 2) Promoting understanding.

**Conclusions:**

The HCPs and participants in this study had a positive experience or viewed the implementation of periodic CGM with data visualization tools as a positive experience and appeared to be feasible for implementation in a primary care setting.

## 1 Introduction

Effective diabetes management necessitates the comprehensive monitoring of glucose levels using various methods that are currently available, including continuous glucose monitoring (CGM). CGM, when used in conjunction with self-monitoring of blood glucose (SMBG) offers a more comprehensive evaluation of glucose control [1]. The FreeStyle Libre system (Abbott Diabetes Care, Witney, Oxon, UK) measures glucose levels in the interstitial fluid every minute and stores the data every 15 min. The sensor is worn for a duration of 14 days [2].

Previous studies on CGM have demonstrated clinical benefits in people with type 2 diabetes receiving insulin therapy [3,4]. A previous randomized controlled trial in adults with type 2 diabetes treated with intensive insulin therapy found no significant differences in hemoglobin A_1c_ (HbA_1c)_ levels after 6 months of CGM or SMBG treatment. However, a subgroup analysis of patients aged <65 years revealed a significant reduction in mean change of HbA_1c_ from baseline in the CGM group. Reduced time in nocturnal hypoglycemia and higher treatment satisfaction were also observed in this group [5]. A recent review of patients with type 2 diabetes reported improved well-being, decreased disease burden, and higher treatment satisfaction with CGM than with SMBG [6]. In another randomized controlled trial, CGM significantly improved the HbA_1c_ levels and treatment satisfaction, without increasing hypoglycemic exposure. These findings suggest that CGM can introduce clinically significant changes in HbA_1c_ when used in primary care settings [7]. Lameijer et al. showed that high HbA_1c_ at baseline predicts significant HbA_1c_ reduction during CGM use [8].

To maximize the benefit of a CGM system, the proficient management of glucose values and trends is essential [9,10]. Utilizing standardized data management programs for the analysis of the CGM data promotes collaboration and provides an opportunity for shared decision support between the health care professionals (HCPS) and individuals with type 2 diabetes. This collaborative approach facilitates the identification of problematic areas and the establishment of achievable goals related to glucose control, treatment and self-care [11,12]. Real-time CGM sensors provide individuals with diabetes immediate access to recorded data. This technology enables individuals with diabetes to monitor glucose dynamics and ensures timely detection of even asymptomatic high- or low-glucose events, that are difficult to capture with SMBG. Time in range (TIR) is a useful metric of glycemic control and glucose patterns [13]. A recent study highlighted a significant association between less time spent on TIR and the prevalence of peripheral neuropathy [14]. The large amount of data obtained from patients with diabetes makes artificial intelligence (AI) techniques particularly attractive for use in support systems and advanced diabetes management [15].

HCPs play a central role in supporting adults with type 2 diabetes through the integration of technology to enhance self-care. Technology should be introduced in a manner that prevents it from being perceived as a burdensome. The optimal use of such technologies for diabetes treatment relies on patients receiving specific training and diabetes education, with HCPs adopting individualized approaches to diabetes care [16]. Although CGM can be useful for HCPs in developing treatment plans and making therapeutic adjustments, its optimal impact on outcomes is realized when individuals living with diabetes actively engage with it to generate positive health benefits [17].

Patients with diabetes highly appreciate HCPs who not only demonstrate professionalism but also provide personalized care [18]. Furthermore, empathy, expert knowledge of diabetes, recognition of the challenges in managing the condition, and understanding the social and emotional impact of the disease are particularly valued. Therefore, providing education and support for HCPs is crucial to delivering an empathic, tailored, person-centered approach to routine diabetes care [18].

Furthermore, health care organizations must take initiatives to integrate psychosocial support seamlessly into routine diabetes care [19]. Schultz et al. identified the key elements that constitute a useful diabetes consultation from the patient's perspective [20]. This study emphasized the importance of adopting a person-centered approach in terms of content and style. Achieving this involves shared responsibility between the HCP and patient. Position statements from the American Diabetes Association and the European Association for the Study of Diabetes advocate person-centered communication, including patient views, and promoting the provider–patient partnership with a more participatory style [21]. Addressing psychosocial and other patient perspectives can be facilitated through the use of digital patient-reported outcomes and experience measures before and during counseling [22,23].

Since 2018, the Swedish National Board of Health and Welfare has recommended CGM for patients with type 2 diabetes receiving multiple daily insulin injections. A recent retrospective real-world study, conducted in a large cohort of patients with type 2 diabetes based on the Swedish National Diabetes Register, showed that patients with type 2 diabetes using the FreeStyle Libre system experienced a sustained reduction in HbA1c levels for at least 12 months after initiation [24]. However, the role of periodic CGM for those not using insulin remains unclear, necessitating additional data to enhance the management type 2 diabetes [4].

Therefore, this study aimed to evaluate and describe the experiences of using periodic continuous glucose monitoring (CGM) with data visualization tools in patients with type 2 diabetes to foster a person-centered approach in a primary care setting.

## 2 Methods

### 2.1 Study design

This feasibility study used a mixed methods design to collect data.

### 2.2 Participants

Three HCPs (one diabetes nurse and two diabetes physicians) were involved in this study to determine the feasibility of implementing periodic CGM. The HCPs were chosen based on their interest in and ability to implement periodic CGM at the outpatient clinic in Stockholm within the study period. Furthermore, they were selected due to their expertise as seasoned specialists in diabetes care.

Patients (n = 50) who visited the outpatient clinic and fulfilled the following criteria were consecutively recruited for this study: aged ≥18 years, diagnosed with type 2 diabetes, and with a disease duration of at least 2 years. Furthermore, the patients were required to possess a smartphone and computer with Internet access for uploading the glucose data. Fifty participants with type 2 diabetes were selected to reflect the perspectives of the patient population targeted for the implementation of periodic CGM in primary care. Table 1 presents the baseline characteristics of the participants.

**Table 1:** Distribution of participants (n = 50) according to demographic and clinical characteristics.

Characteristic	Values
Age in years, mean (SD), range	61.0 (8.8), 39.0–83.0
Male, n (%)	40 (80)
University education, n (%)	21 (42)
Weight in kg, mean (SD), range	93.1 (19.3), 57.2–144.7
Waist circumference in centimeters, mean (SD), range	106.4 (13.7), 82.0–142.0
Diabetes duration in years, mean (SD), range	7.7 (4.6), 2.0–21.0
HbA_1c_ mmol/mol, mean (SD), range	60.1 (17.0), 38.0–116.0
HbA1c %, mean (SD), range	7.65 (1.5), 5.65–12.75
Medication	
Metformin, n (%)	42 (84)
SGLT2, n (%)	21 (42)
DPP-IV inhibitor, n (%)	2 (4)
GLP-1-analog, n (%)	32 (64)
Insulin, n	11 (22)
Employment status	
Working full time, n (%)	31 (62)
Working part time, n (%)	7 (14)
Retired, n (%)	12 (24)

DPP-IV inhibitor, dipeptidyl peptidase-IV inhibitor; GLP-1-analog; glucagon-like peptide-1 analog; HbA_1c_, hemoglobin A_1c_; SGLT2, sodium/glucose cotransporter 2.

### 2.3 Procedures

**Fig. 1:**
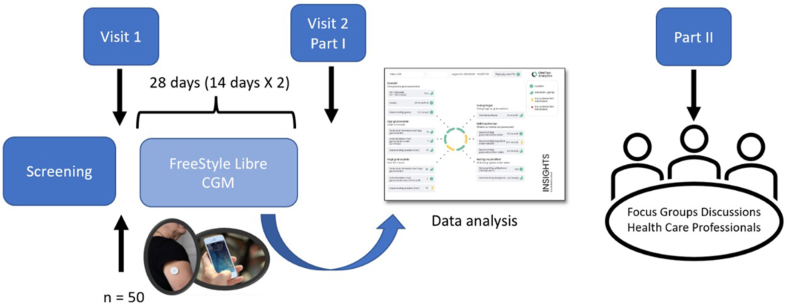
Flowchart showing the feasibility study process.

This study was conducted to assess the feasibility of implementing periodic CGM (a single period of sensor measurements for 28 days) in a primary care setting [25]. The data were collected from April 2021 to January 2022. Initially, a screening was conducted to identify potentially eligible patients with type 2 diabetes from an outpatient clinic in Stockholm (Fig. 1). Subsequently, a diabetes nurse informed the patients about the study and its purpose via phone or video conference. Written research information was also sent by post. Upon confirmation of the willingness to participate in the study, the patient was visited by a diabetes nurse at the outpatient clinic.

All patients provided written informed consent, and the study was conducted in accordance with the Declaration of Helsinki [26] and approved by The Swedish Ethical Review Authority (2021-00792).

During the initial study visit, all patients were instructed on how to apply the 14-day sensor using the applicator, activate the sensor using the FreeStyle LibreLink app, and scan the sensor at least every 8 h. The participants were advised to scan as frequently as desired gain insights from the glucose readings, identify trends and recognize patterns. The patients were advised to maintain records of food intake, medication, physical activity, and other events to enhance their understanding of glycemic readings, patterns, and trends. If symptoms of hypoglycemia or hyperglycemia developed, the patients were instructed to continue performing SMBG with a finger prick.

At the initial visit, the patients were guided on how to log into the National Diabetes Register to access their data. Prior to follow-up (the second study visit), the patients were asked to complete a digital diabetes questionnaire comprising 33 questions about their health and experiences of health care [27,28]. The questionnaire was accessed using a secure login with multi-factor authentication or a one-time password.

Four weeks after the baseline measurement, the first part of the second study visit was conducted at the outpatient clinic. Prior to this visit, the patients completed sensor measurements for 28 days and completed the digital diabetes questionnaire. During the visit, weight, hip circumference, waist circumference, and HbA_1c_ levels were re-measured. Subsequently, the HCPs and patients collaboratively reviewed and discussed trends, patterns, and challenges to support the individual's self-care management of type 2 diabetes. This discussion, along with personalized feedback, was based on the LibreLink data obtained from the CGM device and survey responses regarding feelings and experiences related to diabetes care. This approach is designed to enhance communication and decision-making between the patient and the HCP. The patient data from FreeStyle Libre were transferred to the OneTwo Analytic tool for automated analysis [29]. The OneTwo Analytic tool INSIGHTS helps HCPs and individuals with diabetes in interpreting large amounts of CGM data. This tool employs AI for the advanced automatic analysis of CGM data.

A focus group interview [30] was conducted with the three HCPs involved in the study to discuss their experiences of using periodic CGM with data visualization tools to foster a person-centered approach. U–B J conducted the focus group interview, which was based on a semi-structured interview guide. The discussions. which lasted 40 min, were audio-recorded with a digital voice recorder and subsequently transcribed verbatim. The transcript was analyzed using inductive content analysis [31].

### 2.4 Statistical analysis

No power analysis was conducted since the study was designed as a feasibility study. Instead, a pre-specified sample size of 50 was determined based on convenience sampling and the anticipated need to achieve saturation for the qualitative focus group discussion of the study. Descriptive statistics are reported as mean (standard deviation) or frequency (%) and paired *t*-test was used to compare HbA_1c_ change (baseline to 4 weeks) using IBM SPSS Statistics version 27 (IBM SPSS Inc., Armonk, NY, USA). A p-value of <0.05 was considered significant.

## 3 Results

Fifty individuals with type 2 diabetes were included in this feasibility study. The mean age of the patients was 61.0 (8.8) years, the mean diabetes duration was 7.7 (4.6) years, and 40 % of participants were men (Table 1).

**Fig. 2:**
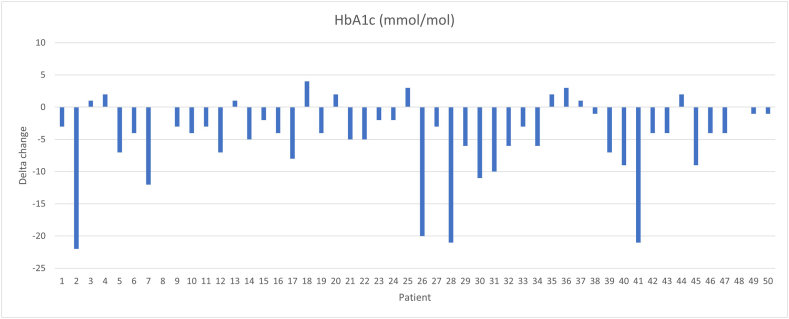
Delta changes in HbA_1c_ levels (mmol/mol) from week 0 (baseline) to 4 weeks.

The participants reported to their HCP that the CGM device was comfortable to wear, and that the LibreView was user-friendly for scanning the sensor to obtain and visualize glucose levels and trends. The participants had no concerns regarding data sharing with the outpatient clinic. The Availability of CGM data was >70 %. Clinical observations revealed a mean reduction in HbA_1c_ from 60.06 mmol/mol [7.65 %] (17.04) at baseline to 55.42 mmol/mol [7.20 %] (12.67) after 4 weeks (p < 0.001). The individual HbA_1c_ delta change from baseline to after four weeks for all participants are presented in Fig. 2.

### 3.1 Results from the focus group discussions

#### 3.1.1 Fostering dialogue on self-care

The HCPs highlighted that the visualization tool LibreView within the FreeStyle Libre system made it easier to discuss diabetes-related issues with patients. The tool's depiction of glucose curves throughout the day, as opposed to specific times or individual blood glucose values, made it easier for HCPs to address specific issues. The HCPs noted that patients, through the tool, gained insights into dietary choices, making it more straightforward for HCP to provide advice and support for self-care. Additionally, the HCPs emphasized that patients' utilization of LibreView, with its glucose reports encompassing patterns and trends, prompted a distinct level of preparedness before their consultations with the HCPs. This preparedness led to more meaningful dialogues about self-care.

Before initiating periodic CGM, HCP experiences patients lacked a full understanding of the necessity to independently track their blood glucose levels. They typically awaited appointments awaited appointments with HCPs to learn about their HbA_1c_ values. The concept of embracing self-care was not ingrained in their approach. However, after experiencing periodic CGM, the patient recognized the significance of self-monitoring and self-care.

HCPs considered the Diabetes Questionnaire a valuable tool for encouraging dialogue with patients. One patient started opened up about previously undisclosed concerns during questionnaire completion. The questionnaire is also a useful conversational tool for patients who may need assistance in initiating discussions. The combination of the visualization tool LibreView and the questionnaire facilitated more accessible conversations about self-care.

#### 3.1.2 Promoting understanding

The visual tool was useful for HCPs as it could show patients' glucose history before and after meals, promptly confirming which meal increased the glucose levels the most. The HCPs described it as opening a door to patients’ understanding. Patients were empowered to interpret the glucose data and comprehend the dynamic of their condition. The HCPs emphasized that the tool was useful not only for patients closer to diagnosis but also for those living with the illness for several years. They emphasized that everyone with type 2 diabetes benefits from periodic CGM, not only patients who receive insulin treatment.

HCPs reported that several patients with long-standing diabetes gained a newfound understanding of how various activities affected their glucose levels only after initiating periodic CGM. The visual tools helped the patients become motivated to adjust their activities, resulting stabilization of their blood glucose levels. The visual tool was also used for care planning by an inter-professional team, and patients appreciated the distinct expertise of each team member.

#### 3.1.3 Illustrative example of dialogue and understanding

One of the three HCPs describes this example.

*A man who had had prolonged high glucose levels and HCP had tried different strategies*. *He was included in the study and started with insulin*. *Then he called me after a week or so and I stop the insulin because the glucose values were stable*. *So*, *he called one more time*, *then he wanted to stop taking the diabetes medication*. *Thereafter*, *came back and had perfectly normal HbA*_*1c*_*values*. *He had learned exactly how to eat and had changed his entire diet*. *And it was great fun to see*. *And this is what he wished he had had from the beginning*. *Because he hadn’t understood how diet and exercise affected his blood sugar*. *Since then, he has continued to buy these sensors himself*. (Told by HCP).

#### 3.1.4 Experiences of OneTwo analytics

HCPs transferred patients’ glucose data from the FreeStyle Libre system to the OneTwo Analytic tool. The HCPs found this process manageable when patients granted permission for data sharing. Furthermore, the HCPs highlighted the educational utility of glucose value bars before and after meals during patient meetings. Recognizing the constraints of limited time during patient meetings, the bars were seen as valuable in streamlining discussions, making them efficient and meaningful. However, HCPs expressed the need for more support and education on how to effectively utilize the OneTwo Analytic tool.

## 4 Discussion

This feasibility study aimed to evaluate and describe experiences of using periodic CGM with data visualization tools in type 2 diabetes to foster a person-centered approach in a primary care setting. This study reported the HCPs’ experience with the protocol and procedures. The results of the study indicated that the protocol and planned procedures worked well together and were feasible, as all participants fulfilled the protocol and planned procedures. Clinical observations during this feasibility study indicated significant beneficial changes in metabolic parameters such as HbA1c levels. A recent retrospective real-world study also showed that individuals with type 2 diabetes using FreeStyle Libre system experienced a reduction in HbA1c levels [24]. Studies on the use of periodic CGM in individuals with type 2 diabetes are lacking. A previous pilot study suggested that cyclic use of real-time CGM could be effective in changing glycemic control, self-care behaviors, and motivation in individuals with type 2 diabetes [32]; the current study adds to the small amount of evidence in this area to date.

Focus group discussions revealed that LibreView in the FreeStyle Libre system fostering dialogue on self-care among individuals with type 2 diabetes and is a visual tool that promoting understanding. Individuals gain insights from patterns and trends in glucose levels that guide decisions about self-care management behaviors, such as physical activity, healthy eating and taking prescribed medication. This experiential learning has been described in a previous study, in which participants commented that seeing the glucose values from the CGM device led to a deeper understanding of the effects of their behaviors on glucose levels [33]. The visual tool LibreView and the glucose reports could be considered valuable tools for preparation before consultations with HCPs. A previous study highlighted the importance of using a preparation tool for patients with diabetes to enable them to express their needs and expectations in consultations with HCPs [20].

HCPs play an important role in supporting individuals with type 2 diabetes to receive and use new technology for diabetes management [16]. Self-management is also an important aspect of diabetes care. This includes collaborating with HCPs, making informed decisions, solving problems, developing personal goals and action plans, and coping with emotions and life stresses [34].

The HCPs in the present study emphasized that periodic CGM and use of the diabetes questionnaire helped patients to prepare for meetings and initiate dialogue about their personal needs with their HCPs. Discussions between members of the HCP team that manage patients are another important component of diabetes care. Promoting the provider–patient partnership and using a more participatory style are important [21] to facilitates person-centered dialogue [23,35].

The HCPs in this study expressed the need for more support and education regarding the use of OneTwo Analytic tool. This assistance should be planned before the initiation of a study start or before its implementation in clinical care and it could be facilitated through digital means. This finding is in line with that of another study, which highlighted that specific training is required for the optimal use of technologies [16].

## 5 Limitations

The study has some limitations. The current study design is a feasibility study. Thus, the results should not be interpreted as empirical evidence for effectiveness of CGM in adults with type 2 diabetes. The study was conducted at only one outpatient clinic and with periodic CGM for 28 days and a follow-up period of 1 month. Further research is needed to evaluate the effectiveness of periodic CGM in type 2 diabetes in a primary care setting.

## 6 Conclusion

The HCPs and participants in this study had a positive experience or viewed the implementation of periodic CGM with data visualization tools as a positive experience and appeared to be feasible for implementation in a primary care setting.

## Funding

This study was supported by the 10.13039/501100023299Sophiahemmet Foundation and 10.13039/501100008546Swedish Diabetes Foundation (DIA2021-66).

## Data availability statement

The authors do not have permission to share data, and the datasets because of the risk of identifying of the study participants.

## CRediT authorship contribution statement

**Unn-Britt Johansson:** Writing – review & editing, Writing – original draft, Funding acquisition, Formal analysis, Data curation, Conceptualization. **Sissel Andreassen Gleissman:** Writing – review & editing, Writing – original draft, Supervision, Methodology, Formal analysis, Data curation. **Maarit Korkeila Lidén:** Writing – review & editing, Writing – original draft, Conceptualization. **Marie Wickman:** Writing – review & editing, Writing – original draft, Conceptualization. **Berit Gustafsson:** Writing – review & editing, Writing – original draft, Project administration, Conceptualization. **Stefan Sjöberg:** Writing – review & editing, Writing – original draft, Conceptualization.

## Declaration of competing interest

The authors declare that they have no known competing financial interests or personal relationships that could have appeared to influence the work reported in this paper.
