# Supplementary material for: Mixed methods study on the feasibility of implementing periodic continuous glucose monitoring among individuals with type 2 diabetes mellitus in a primary care setting
Source: Heliyon. 2024 Apr 16;10(8):e29498. doi: 10.1016/j.heliyon.2024.e29498 (PMC11041009; doi:10.1016/j.heliyon.2024.e29498)
Supplement: Multimedia component 1 [file mmc1.docx]

**Interview guide used for focus group discussion with health care professionals**

- What experiences do you have regarding using periodic continuous glucose monitoring for persons with type 2 diabetes?
- What is your view on the usefulness of using continuous glucose monitoring for persons with type 2 diabetes?
- During the appointment, you had a dialogue with the patient about the interpretation of glucose data based on the digital data visualization tool LibreView from FreeStyle Libre.

What benefits do you see in using the digital data visualization tool in the care meeting to interpret glucose values and optimize treatment as well as to support the patient's self-care management?

- What experience do you have regarding the use of digital data visualization tools to provide conditions for discussion on interpreting trends, patterns and challenges to support the person's self-care management?
- Do you see any disadvantages of using the digital data visualization tool in the healthcare meeting?
- In what way can the digital visualization tool be used in healthcare meetings?
- You downloaded data from the FreeStyle Libre system to the OneTwo Analytic tool for automated analysis.

How user-friendly did you experience the process of downloading data?

How user-friendly did you think the digital visualization tool was for OneTwo Analytic tool?

- What experience do you have regarding the use of digital data visualization tools to provide conditions for discussion on interpreting trends, patterns and challenges to support the person's self-care management?
- What features of OneTwo Analytic tool did you like the best?
- What features of OneTwo Analytic tool did you dislike?

- Have you other thoughts about OneTwo Analytic tool?
- Is there anything else you would like to add?
